# Association of remnant cholesterol with cardiovascular events and mortality in biopsy-proven diabetic kidney disease

**DOI:** 10.3389/fendo.2026.1720189

**Published:** 2026-01-28

**Authors:** Qiyuan Hu, Ling Chen, Lingzhi Xing, Yamei Xu, Jiachuan Xiong, Yuewu Tang

**Affiliations:** 1 The Second Affiliated Hospital, Chongqing Medical University, Chongqing, China; 2 The Center of Experimental Teaching Management, Chongqing Medical University, Chongqing, China; 3 Faculty of Pediatrics, Chongqing Medical University, Chongqing, China; 4 Department of Nephrology, The Key Laboratory for the Prevention and Treatment of Chronic Kidney Disease of Chongqing, Chongqing Clinical Research Center of Kidney and Urology Diseases, Xinqiao Hospital, Army Medical University (Third Military Medical University), Chongqing, China; 5 Department of Nephrology, Chongqing University Three Gorges Hospital, Chongqing, China

**Keywords:** cardiovascular disease, diabetic kidney disease, mortality, remnant cholesterol, risk factor

## Abstract

**Background:**

Remnant cholesterol (RC) has been recognized as a critical risk factor for vascular diseases. However, its association with cardiovascular disease (CVD) and overall mortality in individuals with diabetic kidney disease (DKD) has not been thoroughly investigated.

**Methods:**

This retrospective single-center study enrolled 329 patients with biopsy-confirmed DKD from August 2009 to December 2018. The prognosis of patients with varying RC levels was compared. In addition, the association between RC levels and left ventricular structure and function was examined. Furthermore, the predictive capability of RC for clinical outcomes was assessed.

**Results:**

Over the follow-up period, 76 patients (23.1%) experienced CVD events, while 44 patients (13.4%) died. Kaplan-Meier analysis demonstrated that patients in the high RC group exhibited significantly elevated rates of CVD events (*p* = 0.0047) and all-cause mortality (*p* = 0.0213). Additionally, multivariate Cox regression analysis confirmed RC as an independent risk factor for CVD events (HR = 1.323, 95% CI 1.076-1.626, *p* = 0.008) and overall mortality (HR = 1.359, 95% CI 1.039-1.779, *p* = 0.025). Finally, the AUC analysis indicated that the inclusion of RC in traditional risk factors improved their predictive accuracy.

**Conclusion:**

RC independently contributes to the risk of CVD events and all-cause mortality in individuals with biopsy-confirmed DKD.

## 1 Introduction

Diabetic kidney disease (DKD) refers to chronic kidney disease (CKD) attributable to diabetes mellitus (DM), characterized by persistent proteinuria and a progressive decline in renal function (1). Its prevalence has surged to alarming levels, rendering DKD a pressing public health issue. Cardiovascular disease (CVD) encompasses a broad spectrum of clinical entities, including atherosclerotic cardiovascular disease (ASCVD) as well as heart failure, which collectively represent the major causes of disability and death in patients with DM and CKD (2, 3). Frequently coexisting with arterial hypertension, DKD intensifies the burden of morbidity and mortality attributable to CVD (4). Significantly, research has demonstrated that individuals with type 1 (T1DM) or type 2 (T2DM) diabetes mellitus who develop DKD exhibit markedly elevated rates of CVD events and mortality in comparison to those without DKD (1). Beyond their epidemiological co-occurrence, the cardiovascular risk in patients with DKD is also reflected in the interconnected pathophysiological processes linking the heart and kidneys. Albuminuria and reduced glomerular filtration rate are considered systemic markers of endothelial dysfunction and diffuse microvascular injury, which are closely associated with the development and progression of atherosclerotic disease (5–7).

In line with this clinical framework, the KDIGO guidelines on diabetes management in CKD (2020 and the 2022 update) emphasize risk-stratified management based on eGFR and albuminuria categories, and recommend a comprehensive strategy centered on renin–angiotensin–aldosterone system (RAAS) inhibition, sodium–glucose cotransporter 2 (SGLT2) inhibitors, and nonsteroidal mineralocorticoid receptor antagonists to reduce kidney disease progression and cardiovascular events, thereby providing an international evidence-based foundation for long-term DKD management (8, 9).

Concurrently, neurohormonal activation, particularly the upregulation of the renin-angiotensin-aldosterone system, leads to increased arterial stiffness, vascular remodeling, and adverse cardiac structural changes, thereby elevating the risk of CVD events (10, 11). Furthermore, chronic low-grade inflammation, oxidative stress, insulin resistance, and the accumulation of uremic solutes in DKD exacerbate vascular dysfunction and accelerate atherogenesis (12, 13). Collectively, these mechanisms provide a pathophysiological rationale for the substantial CVD burden in DKD patients and underscore the necessity for more precise risk management in this population (14).

Previous studies have demonstrated the early onset of dyslipidemia in diabetes, persisting as an independent risk factor for DKD (15). Within the spectrum of lipoprotein profiles, low-density lipoprotein cholesterol (LDL-C) emerges as particularly significant (16, 17). Traditionally, the primary focus of CVD prevention has been the reduction of LDL-C levels through widespread statin use (18). However, research indicates that patients with markedly reduced LDL-C levels still exhibit a substantial residual risk for CVD (19). Given the ineffectiveness of treatments aimed at increasing high-density lipoprotein cholesterol (HDL-C) in reducing CVD, attention has shifted to another marker of dyslipidemia—triglyceride (TG)-rich lipoproteins (TRLs) that statins are unable to regulate (20, 21). Numerous studies have provided substantial evidence linking TRLs to the development of CVD (22, 23). Importantly, most cells efficiently metabolize TG, prompting the hypothesis that the detrimental component in TRLs is cholesterol, which is more prone to accumulation in the body (24).

TRLs include very low-density lipoproteins (VLDL), intermediate-density lipoproteins (IDL), and chylomicron (CM) remnants. Remnant cholesterol (RC) refers to the cholesterol content within these TRLs (25). The measurement of RC can be achieved directly (RC=TC-HDL-LDL) or estimated by dividing the serum total triglycerides (in mg/dL) by 5 (24). Several studies have established an association between RC and atherosclerotic heart disease in both primary and secondary prevention of CVD, suggesting that RC may indeed independently contribute to the prevention of CVD (26–28).

Preliminary evidence suggests that RC may have some predictive capacity for the progression of DKD and diabetic retinopathy (DR) (29). However, the predictive value of RC for CVD and mortality in DKD patients has yet to be evaluated. Concurrently, RC has been observed to impact left ventricular (LV) structure and function, further suggesting its potential contribution to cardiovascular risk (30). Therefore, in this retrospective cohort study, we aimed to evaluate whether baseline serum RC is independently associated with subsequent CVD events and all-cause mortality among patients with DKD, and to assess whether adding RC to models including established risk factors improves risk prediction for these outcomes.

## 2 Materials and methods

### 2.1 Study design

This retrospective study analyzed 329 patients diagnosed with DKD confirmed by biopsy at Xinqiao Hospital of the Army Medical University in China between August 2009 and December 2018. The diagnosis of DKD was based on the criteria established by the Renal Pathology Society in 2010 (31). Participants were monitored from the date of screening until January 1, 2019, or until their death. Inclusion criteria comprised (1): biopsy-confirmed diabetic nephropathy, encompassing both diabetic nephropathy alone and diabetic nephropathy combined with nondiabetic nephropathy (2); individuals aged 18 years or older; and (3) comprehensive medical records and follow-up data. Exclusion criteria included (1): occurrence of endpoint events within 1 month of patient enrollment (2); incomplete pathological or blood analysis results (3); patients diagnosed with malignant tumors; and (4) patients with RC levels less than or equal to zero. The study protocol received approval from the ethical committee of Xinqiao Hospital (No. 2018-006-02).

### 2.2 Data collection

Baseline demographic characteristics and laboratory values were retrieved from the Electronic Medical Record System (EMRS) of Xinqiao Hospital at the time of the patient’s initial renal biopsy, encompassing (1): demographic data, such as age and gender (2); medical history, including hypertension, diabetes mellitus, and coronary artery disease (3); physical examination data, such as height, weight, and systolic/diastolic blood pressure; and (4) laboratory information, such as lymphocyte count, hemoglobin, serum creatinine, blood urea (BUN), uric acid, parathyroid hormone (PTH), calcium, magnesium, phosphate, albumin, total cholesterol (TC), LDL-C, HDL-C, TG, proteinuria, and pathological details. The estimated glomerular filtration rate (eGFR) was computed using the Chronic Kidney Disease Epidemiology Collaboration formula. Body mass index (BMI) was calculated by dividing the weight (in kilograms) by the square of the height (in meters). Pathological lesions were routinely assessed by renal pathologists using light and electron microscopy in accordance with the 2010 RPS criteria (31). Furthermore, RC was computed based on three laboratory variables: TC, HDL-C, and LDL-C, with RC = TC – HDL-C – LDL-C (24).

### 2.3 Definition and ascertainment of DKD

DKD was ascertained by a kidney biopsy. All biopsy specimens were evaluated by the Department of Pathology at our hospital, and DKD was diagnosed according to standard histopathological criteria for diabetic nephropathy (31). Patients were included if they had a clinical diagnosis of diabetes and a kidney biopsy confirming DKD. Patients were excluded if the biopsy did not support DKD as the primary diagnosis, if there was a predominant alternative renal pathology, or if biopsy/clinical data required for classification were unavailable.

### 2.4 Clinical outcomes

The study evaluated two outcomes: CVD events and all-cause mortality, each considered independently. CVD events encompassed the onset of new CVD occurrences, including coronary heart disease, heart failure, cerebrovascular events, and severe arrhythmia. All-cause mortality was defined as death resulting from any cause. Clinical outcomes in this study were primarily obtained through telephone follow-up or patient medical record reports.

### 2.5 Echocardiography measurements

Echocardiographic measurements were conducted in accordance with standardized operational criteria. To assess LV structure, the left ventricular mass index (LVMI) and relative wall thickness were determined. LVMI was computed as the LV mass derived from the Devereux formulation indexed to the body surface area, which was estimated using the Du Bois formula. Relative wall thickness was calculated by dividing the posterior wall thickness by the LV internal dimension in diastole. To evaluate LV systolic function, the LV ejection fraction was measured. For LV diastolic function assessment, the early peak diastolic mitral inflow velocity (E) and early peak diastolic mitral annular velocity (e′) were examined. The E/e′ ratio, a measure of LV filling pressure, was computed by dividing E by e′.

### 2.6 Statistical analysis

The Kolmogorov-Smirnov test was employed to evaluate the normal distribution of all data. Normally distributed data were expressed as mean ± standard deviation, while non-normally distributed data were presented as median (interquartile range). Group differences were assessed using the t-test, Mann-Whitney U test, and chi-square test. Spearman’s rank correlation analysis was utilized to examine the relationship between RC and selected characteristics or parameters. Furthermore, Kaplan-Meier curves were employed to compare the prognosis of patients based on RC. Subgroup analyses were conducted based on different clinical characteristics to evaluate the potential impact of RC on specific population segments. Additionally, nested univariate and multivariate Cox proportional risk models were constructed to assess the independent associations between RC and CVD events as well as all-cause mortality, calculating hazard ratios (HRs) and 95% confidence intervals (CIs). The adjusted models were as follows: Model 1: adjusted age, gender; Model 2: adjusted covariates in model 1 plus diabetic retinopathy; Model 3: adjusted covariates in model 2 plus eGFR, serum creatinine, cystatin C, calcium, albumin, lipoprotein(a), interstitial fibrosis and tubular atrophy (IFTA), interstitial inflammation, arteriolar hyalinosis, neutrophil, lymphocyte, proteinuria; Model 4: adjusted covariates in model 3 plus RC. Subsequently, nomograms were utilized to evaluate the effect of each influencing factor on the probability of outcome events based on multifactorial Cox regression models, which in turn calculated the predicted value of the outcome event for each individual. Meanwhile, the predictive ability of RC for clinical endpoint events was evaluated using time-dependent receiver operating characteristic (td-ROC) curves, and the area under the curve (AUC) was calculated for different models. All analyses were performed using SPSS (version 25.0) or R software (version 4.1.3), and a bilateral *p*-value < 0.05 was considered statistically significant.

## 3 Results

### 3.1 Baseline features of the patients

The study included a total of 329 patients with DKD for analysis (**Supplementary Figure 1**). The mean age of the participants was 51 years (IQR: 45-58), with 37.7% (124) being female. Among the patients, 16.1% (53) had hypertension, and 16.1% (53) had a history of coronary heart disease. Additionally, 17.95% (59) used statins, 38.3% (126) received antiplatelet agents, and 12.2% (40) were administered anticoagulants. Meanwhile, glycemic control was achieved with oral hyperglycemic agents in 30.1% (99), while 31.0% (102) were treated with insulin. The median eGFR and proteinuria were 65.63 mL/min/1.73 m^2^ (IQR: 38.23-99.07) and 2.47 g/day (IQR: 0.74-5.15), respectively. Furthermore, 33.7% (111) of patients reported being smokers. The median RC level was 0.70 (IQR: 0.40-1.10). The baseline demographic and clinical characteristics of the study population were presented in **Table 1**. Additionally, all patients’ renal pathology parameters were analyzed (**Supplementary Table 1**).

**Table 1:** Clinical features of the patients stratified across the tertiles of RC.

Variable	All (n = 329)	Tertile1 (n = 110)	Tertile 2 (n = 110)	Tertile 3 (n = 109)	*P-*value
RC, mmol/L	0.70 (0.40-1.10)	0.28 (0.20-0.40)	0.7 (0.60-0.77)	1.29 (1.10-1.93)	<0.001
Female, %	124 (37.7)	42 (38.1)	39 (35.5)	43 (39.4)	0.823
Age, years	51.08 ± 10.41	51.06 ± 10.98	50.62 ± 10.41	51.48 ± 9.88	0.831
Proteinuria, g/day	2.47 (0.74-5.15)	1.90 (0.64-4.28)	2.26 (0.68-4.51)	2.97 (0.90-6.34)	0.059
Smoking, %	111 (33.7)	33 (30)	43 (39.1)	35 (32.1)	0.328
BMI	25.03 ± 3.30	24.61 ± 3.00	25.03 ± 3.23	25.47 ± 3.60	0.151
Hypertension, %	225 (68.4)	81 (73.6)	74 (67.3)	70 (64.2)	0.310
Blood glucose, mmol/L	6.90 (5.40-9.21)	6.98 (5.49-9.56)	6.60 (4.82-8.56)	7.30 (5.50-9.25)	0.402
HBA1C, mmol/L	7.30 (6.50-8,95)	7.30 (6.30-9.23)	7.35 (6.80-8.63)	7.40 (6.45-8.85)	0.644
Coronary heart disease, %	53 (16.1)	17 (15.5)	17 (15.5)	19 (17.4)	0.900
Diabetic retinopathy, %	185 (56.2)	61 (55.5)	66 (60)	58 (53.2)	0.587
White blood cell, ×10^12^/L	6.82 ± 1.92	6.62 ± 1.69	6.92 ± 2.24	6.92 ± 1.79	0.398
Neutrophil, %	66.70 ± 9.98	65.17 ± 9.78	63.89 ± 11.22	64.94 ± 8.84	0.615
Lymphocyte, %	24.82 ± 8.21	24.69 ± 8.33	24.92 ± 8.59	24.85 ± 7.74	0.978
Hemoglobin, g/L	115 (96-135)	110 (91-131)	115 (98-133.5)	120 (103.5-145)	0.068
Platelet counts, ×10^9^/L	199 (157-244)	192.5 (157-234.5)	210.5 (160.5-262.5)	187 (157-235.5)	0.185
Red blood cell, ×10^12^/L	4.00 (3.43-4.54)	3.88 (3.16-4.31)	4.00 (3.49-4.55)	4.00 (3.62-4.72)	0.060
eGFR, mL/min/1.73m^2^	65.63 (38.23-99.07)	63.35 (35.62-99.60)	65.31 (36.48-98.00)	67.00 (39.55-99.35)	0.943
Serum creatinine, umol/L	109.00 (70.95-154.35)	109.70 (69.47-158.97)	108.75 (69.25-154.17)	108.40 (71.40-149.60)	0.811
Uric acid, umol/L	382.54 ± 106.08	378.86 ± 102.02	379.42 ± 112.71	389.39 ± 103.82	0.712
BUN, mmol/L	7.24 (5.52-9.86)	7.34 (5.48-10.12)	7.01 (5.31-9.76)	7.25 (5.71-9.93)	0.460
Cystatin C, mg/L	1.46 (1.03-2.10)	1.50 (0.90-2.04)	1.36 (1.04-2.07)	1.51 (1.04-2.31)	0.966
Calcium, mmol/L	2.19 (2.05-2.31)	2.18 (2.05-2.28)	2.20 (2.08-2.32)	2.18 (2.04-2.32)	0.262
Phosphorus, mmol/L	1.16 (1.02-1.34)	1.13 (1.01-1.31)	1.15 (1.03-1.34)	1.18 (1.03-1.33)	0.579
Parathyroid hormone, pg/ml	64.30 (39.45-90.60)	64.30 (42.38-114.03)	56.75 (40.00-72.98)	59.10 (34.95-95.50)	0.023
Albumin, g/L	35.20 (27.95-41.85)	36.50 (29.60-42.43)	34.80 (27.45-40.80)	34.16 (27.00-41.90)	0.273
Total cholesterol, mmol/L	5.20 (4.22-6.40)	4.22 (3.59-5.00)	5.33 (4.51-6.39)	6.21 (5.29-7.63)	<0.001
Triglyceride, mmol/L	1.70 (1.19-2.65)	1.25 (0.99-1.87)	1.64 (1.20-2.31)	2.49 (1.68-3.86)	<0.001
Lipoprotein (a), mmol/L	248.0 (109.0-588.5)	198.5 (81.0-531.5)	127.5 (249.0-530.5)	312.0 (117.0-703.0)	0.027
HDL, mmol/L	1.17 (0.94-1.46)	1.15 (0.93-1.45)	1.18 (0.93-1.55)	1.18 (0.96-1.46)	0.883
LDL, mmol/L	3.09 (2.49-4.01)	2.74 (2.29-3.29)	3.36 (2.63-4.24)	3.44 (2.50-6.80)	<0.001
C-reactive protein, mg/L	4.40 (2.35-6.80)	4.45 (2.20-6.80)	4.30 (2.38-6.80)	4.40 (2.50-6.80)	0.940
Statin use, %	59 (17.9)	17 (15.5)	23 (21.3)	19 (17.1)	0.377
Anticoagulant use, %	40 (12.2)	12 (10.9)	13 (12.0)	15 (13.5)	0.194
Oral hypoglycemic agents, %	99 (30.1)	31 (28.2)	34 (30.9)	34 (31.2)	0.888
Insulin, %	102 (31.0)	21 (28.2)	38 (34.5)	33 (30.3)	0.585
Antiplatelet drugs, %	126 (38.3)	40 (36.4)	47 (42.7)	39 (35.8)	0.514

RC, remnant cholesterol; BMI, body mass index; eGFR, estimated glomerular filtration rate; BUN, blood urea nitrogen; HDL, high-density lipoprotein; LDL, low-density lipoprotein. IQR, interquartile range. Data are presented as mean ± SD, N (%) or median (IQR).

**Figure 1:**
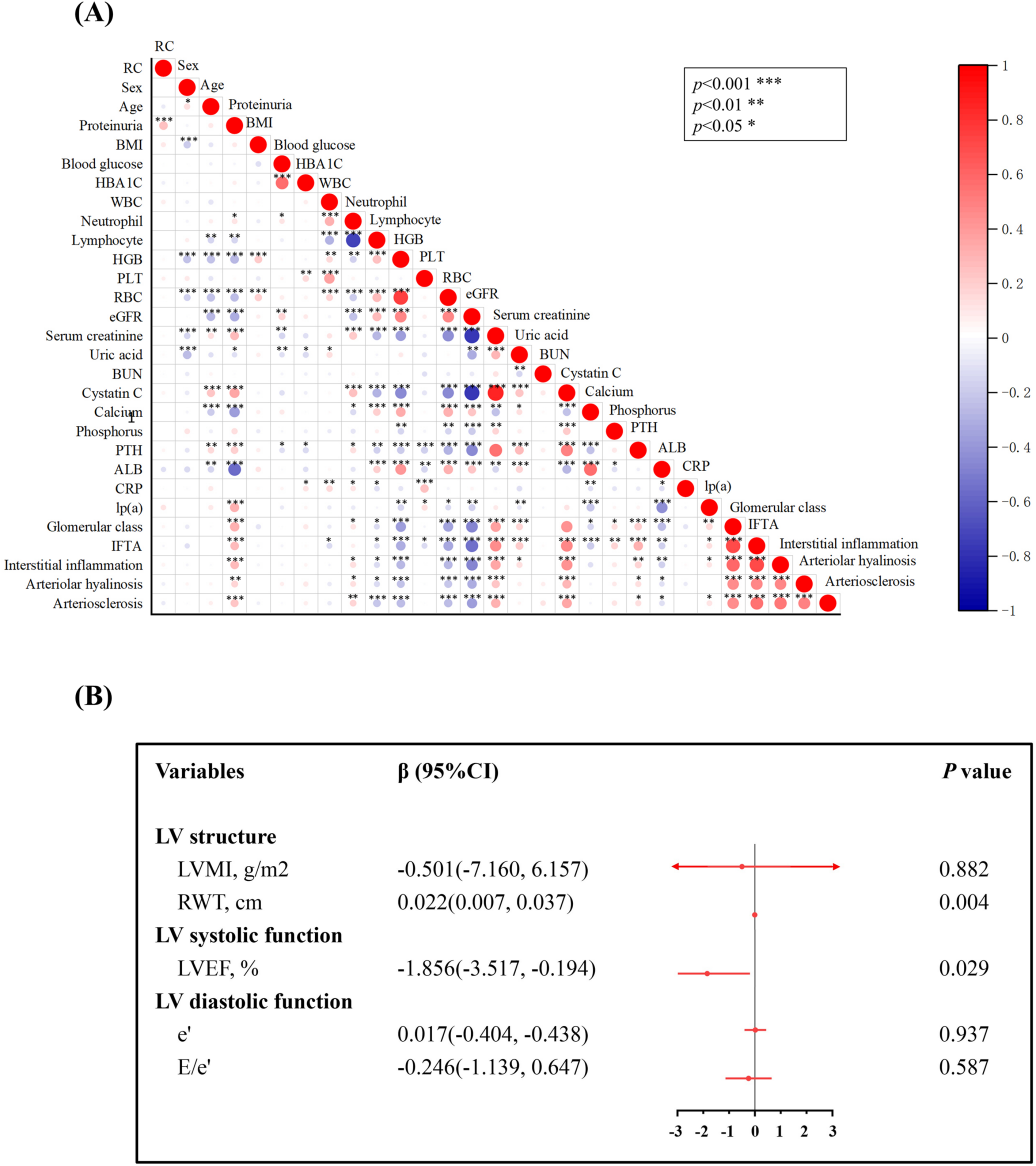
The correlation between remnant cholesterol (RC) and clinical, pathological parameters**(A)** as well as left ventricular (LV) structure and function **(B)**. Models were adjusted for baseline covariates: age, sex, diabetic retinopathy, hypertension, coronary heart disease, smoking, proteinuria, eGFR, serum creatinine, hemoglobin, red blood cell, calcium, albumin and triglyceride. LVMI, left ventricular mass index; RWT, relative wall thickness. LVEF, left ventricular ejection fraction; e′ denotes early peak diastolic mitral annular velocity; E/e′, early peak diastolic mitral inf velocity to early peak diastolic mitral annular velocity. Data are presented for change in LV parameter per 1 unit in RC.

Based on their RC levels, all patients were categorized into three groups: tertile 1 (RC < 0.50), tertile 2 (0.50 ≤ RC ≤ 0.92), and tertile 3 (RC > 0.92). It was observed that individuals with higher RC exhibited elevated levels of total cholesterol, triglycerides, lipoprotein(a), and LDL-C compared to those with lower RC. However, there were no discernible differences in gender, age, BMI, smoking status, hypertension, coronary heart disease, diabetic retinopathy, statin use, or anticoagulant use across the varying RC tertiles. Subsequently, no significant differences were noticed in pathological classification, interstitial inflammation, arteriolar hyalinosis, or atherosclerosis in the three groups. Next, we explored the correlation between RC and other clinical and pathological parameters. The correlation analysis demonstrated that RC was positively associated with proteinuria (r = 0.249, *p* < 0.001), total cholesterol (r = 0.551, *p* < 0.001), and triglycerides (r = 0.596, *p* < 0.001) in terms of clinical characteristics (**Figure 1**). Additionally, the study investigated the impact of RC on LV structure and function. Linear regression analysis was employed to assess the relationship between RC and echocardiographic indices in DKD patients. After adjusting for covariates, higher RC levels were positively correlated with increased relative wall thickness (β = 0.022; *p* = 0.004), despite no association being observed between RC and LV mass index. Regarding LV systolic function, higher RC was significantly correlated with left ventricular ejection fraction (LVEF) (β = -1.856; *p* = 0.029) in fully adjusted models (**Figure 1**). However, no correlation was observed between RC and either early peak diastolic mitral annular velocity (e′) or the E/e′ ratio in relation to LV diastolic function.

### 3.2 Association between RC and CVD and mortality

**Figure 2:**
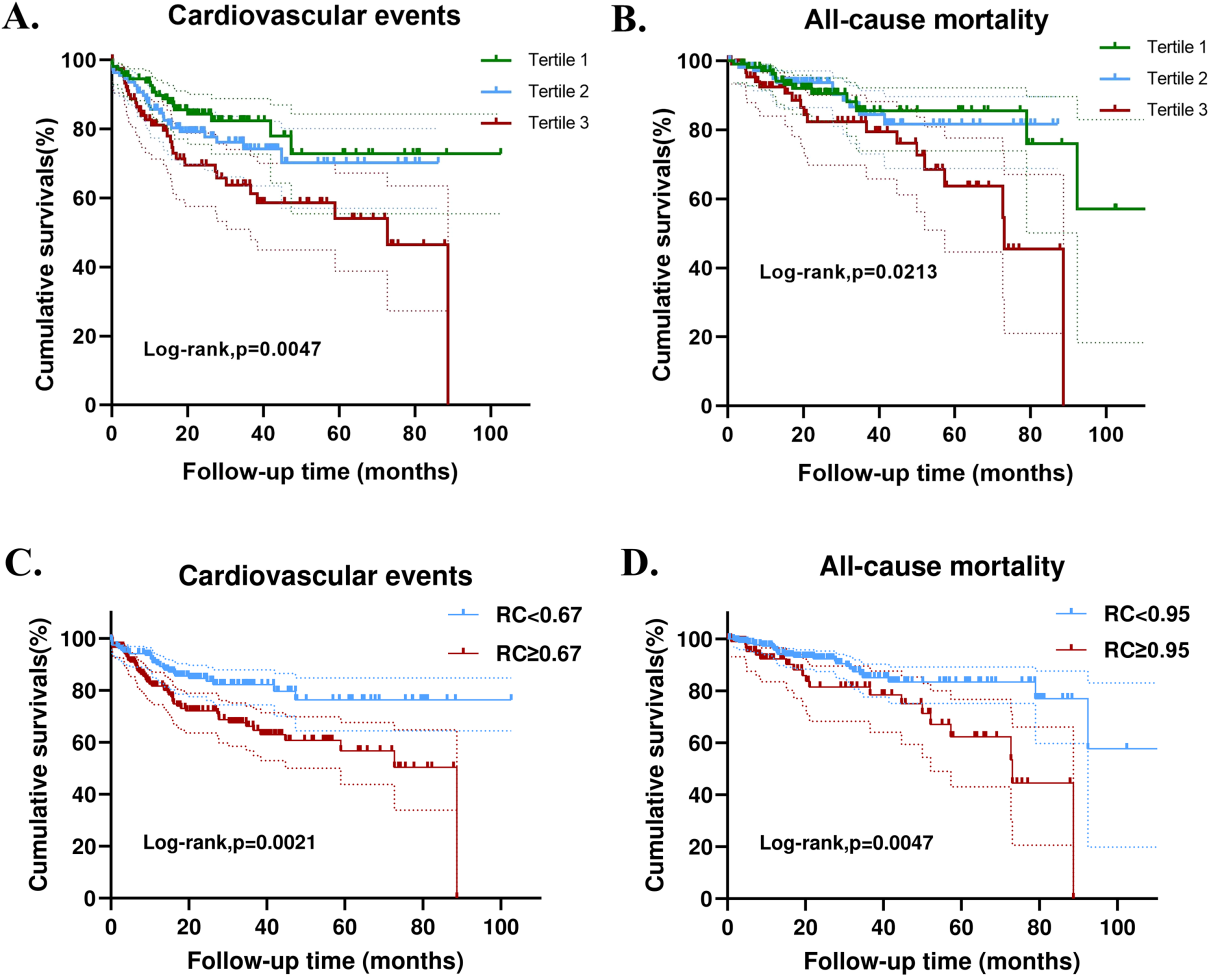
Kaplan-Meier curves for outcomes in patients with biopsy-confirmed DKD across different remnant cholesterol (RC) groups. Divide patients into three groups based on the RC levels: tertile 1 (<0.50), tertile 2 (0.50-0.92), and tertile 3 (>0.92). The upper and lower dashed lines represent the 95% confidence intervals. **(A)** CVD events. **(B)** All-cause mortality. The RC cutoff was identified using time-dependent receiver operating characteristic (td-ROC) curve analysis. Participants were subsequently dichotomized based on this cutoff, and Kaplan–Meier curves were generated accordingly. **(C)** CVD events. **(D)** All-cause mortality.

Over a mean follow-up duration of 31.36 months, 76 patients (23%) developed CVD events, and 44 patients (13.4%) died. The Kaplan-Meier curve demonstrated that patients with higher RC had a significantly higher likelihood of experiencing CVD events (p = 0.005) and overall mortality (p = 0.021) compared to those in lower RC groups (**Figures 2A**). Time-dependent receiver operating characteristic (Td-ROC) analysis was subsequently used to identify the optimal RC cutoff values for discriminating CVD events and all-cause mortality, yielding cutoffs of 0.67 for CVD events and 0.95 for all-cause mortality (**Supplementary Figure 2**). Kaplan-Meier analysis based on these cutoff values demonstrated the robust impact of RC levels on the occurrence of CVD events and all-cause mortality (**Figures 2C**).

In the univariate Cox regression analysis, several factors, including age, gender, diabetic retinopathy, eGFR, cystatin C, calcium, albumin, lipoprotein(a), neutrophil count, lymphocyte count, and proteinuria, were identified as potential factors affecting outcomes in patients with biopsy-confirmed DKD (**Table 2**). However, in the multivariate Cox regression analysis, only age, eGFR, and RC were confirmed as independent risk factors for CVD events. Similarly, albumin, lymphocyte count, proteinuria, and RC were identified as independent risk factors for all-cause mortality. Furthermore, a nested Cox proportional hazards model was employed to evaluate the impact of medication use on the predictive ability for clinical outcomes of RC (**Supplementary Table 2**).

**Table 2:** Univariate and multivariate cox analyses on variables for the prediction of endpoint events in DKD patients.

Variable	CVD event				Death			
	Univariate		Multivariate		Univariate		Multivariate	
	HR (95% CI)	*p-*value	HR (95% CI)	*p-*value	HR (95% CI)	*p*-value	HR (95% CI)	*p-*value
Gender	0.893 (0.555-1.438)	0.643			1.504 (0.830-2.726)	0.179		
Age	1.038 (1.014-1.061)	0.001	2.021 (1.234-3.309)	0.005	1.053 (1.020-1.086)	0.001		
eGFR	0.983 (0.976-0.990)	<0.001	0.988 (0.976-1.000)	0.048	0.980 (0.971-0.989)	<0.001		
Diabetic retinopathy	1.647 (1.014-2.676)	0.044			2.769 (1.324-5.790)	0.007		
Cystatin C	1.594 (1.306-1.947)	<0.001			1.796 (1.381-2.335)	<0.001		
Calcium	0.310 (0.097-0.996)	0.049			0.049 (0.011-0.213)	<0.001		
Albumin	0.965 (0.940-0.990)	0.007			0.911 (0.878-0.945)	<0.001	0.930 (0.881-0.981)	0.008
Lipoprotein (a)	1.001 (1.000-1.001)	0.048			1.001 (1.000-1.002)	<0.001		
RC	1.321 (1.088-1.603)	0.005	1.323 (1.076-1.626)	0.008	1.377 (1.068-1.777)	0.014	1.359 (1.039-1.779)	0.025
Neutrophil	1.049 (1.020-1.079)	<0.001			1.078 (1.037-1.120)	<0.001		
Lymphocyte	0.950 (0.921-0.980)	0.001			0.906 (0.869-0.945)	<0.001	0.932 (0.881-0.986)	0.014
Proteinuria	1.108 (1.050-1.168)	<0.001			1.085 (1.004-1.172)	0.039	0.871 (0.761-0.997)	0.044

HR, hazards ration; CI, confidence interval; CVD: cardiovascular diseases; eGFR, estimated glomerular filtration rate; RC, remnant cholesterol.

**Figure 3:**
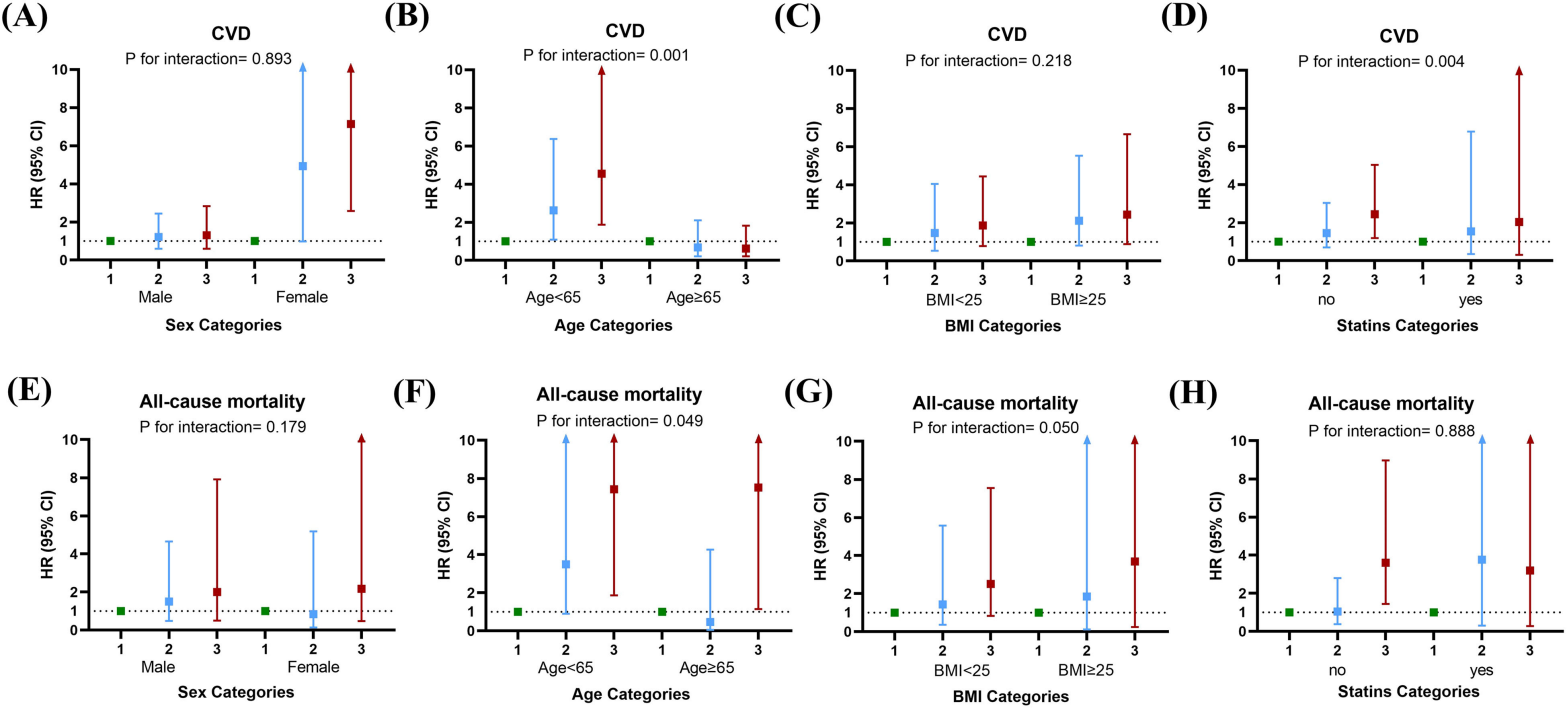
Subgroup analyses of the relationship between remnant cholesterol (RC) levels and clinical outcomes. The study population was stratified by age, sex, BMI and statin use. **(A–D)** CVD events. **(E–H)** All-cause mortality. Adjustments for age, gender, diabetic retinopathy, eGFR, serum creatinine, cystatin C, calcium, albumin, lipoprotein(a), IFTA, interstitial inflammation, arteriolar hyalinosis, neutrophil, lymphocyte, and proteinuria were made.

Next, subgroup analysis was also performed to investigate the association between RC and CVD as well as all-cause mortality. The study population was stratified by age, sex, BMI, and statin use (**Figure 3**). Results indicated an increase in CVD events with higher RC grades regardless of age (≥65 or <65, *p* = 0.001) and with or without statin use (*p* = 0.004). Additionally, the risk of mortality also increased with higher RC grades regardless of age (≥65 or <65, *p* = 0.049) and regardless of BMI (>25 or <25, *p* = 0.050). Sensitivity analyses for subgroup factors demonstrated a significant impact of RC levels on the risk of developing CVD and mortality in women, patients under 60 years of age, obese patients, and patients not taking statins (**Supplementary Figure S2**). Finally, Cox proportional hazards models were utilized to analyze the correlation between RC and CVD events, as well as all-cause mortality. The results suggested that RC remained an independent predictor of CVD events (HR = 1.323, 95% CI 1.076-1.626, *p* = 0.008) and all-cause mortality (HR = 1.359, 95% CI 1.039-1.779, *p* = 0.025) even after multivariable adjustment in different models (**Table 3**).

**Table 3:** Multivariate Cox analysis of endpoint events in patients with DKD.

Endpoint events	Model 1		Model 2		Model 3	
	HR (95% CI)	*p-*value	HR (95% CI)	*p-*value	HR (95% CI)	*p-*value
CVD events						
RC	1.358 (1.117-1.652)	0.002	1.404 (1.146-1.722)	0.001	1.323 (1.076-1.626)	0.008
Death						
RC	1.344 (1.031-1.752)	0.029	1.345 (1.016-1.782)	0.038	1.359 (1.039-1.779)	0.025

RC was analyzed as a continuous variable with hazard ratio (HR). CI, confidence interval.

Model 1: adjusted age, gender;

Model 2: adjusted age, gender, statin, anticoagulant drugs, oral hypoglycemic agents, insulin, antiplatelet drugs;

Model 3: adjusted age, gender, diabetic retinopathy, eGFR, cystatin C, calcium, albumin, lipoprotein(a), neutrophil, lymphocyte, proteinuria.

### 3.3 RC and the prediction of clinical outcomes

**Figure 4:**
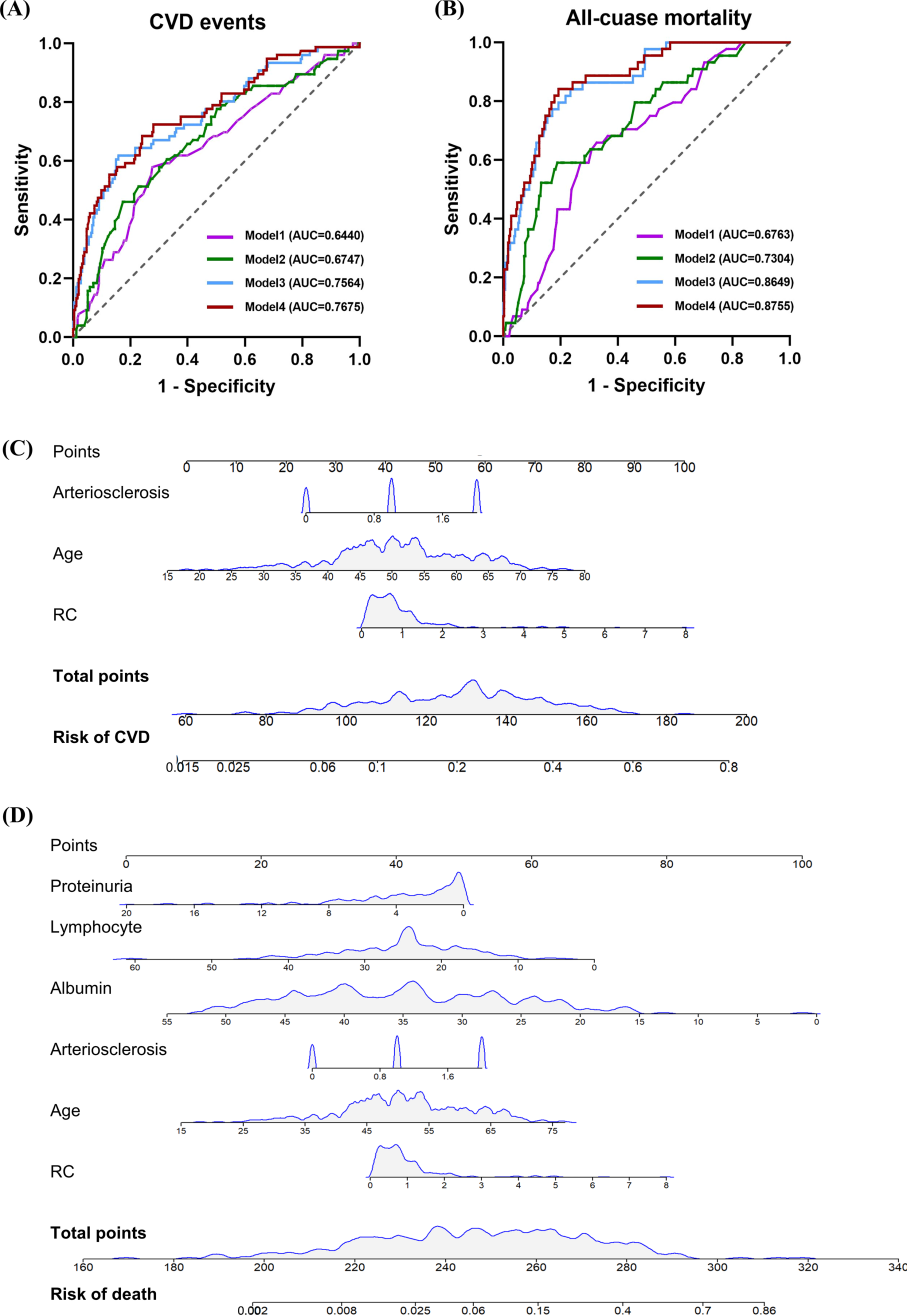
The prediction of RC for clinical outcomes in patients with biopsy-confirmed DKD. The prediction of clinical outcomes by the RC was evaluated using the time-dependent receiver operating characteristic (td-ROC) of the subjects. **(A)** Prediction of CVD events by RC. **(B)** Prediction of all-cause mortality by RC. Nomogram for predicting the risk of CVD**(C)** and all-cause mortality**(D)** in patients with biopsy-confirmed DKD based on multivariate Cox regression analysis.

Finally, we used time-dependent receiver operating characteristic (td-ROC) analysis to analyze the predictive ability of RC for clinical outcomes. For predicting CVD events, the AUC for model 1 (adjusted for age and gender) and model 2 (model 1 plus diabetic retinopathy) were 0.6440 and 0.6747, respectively. Furthermore, after adjusting for additional factors (age, gender, diabetic retinopathy, eGFR, serum creatinine, cystatin C, calcium, albumin, lipoprotein(a), interstitial fibrosis and IFTA, interstitial inflammation, arteriolar hyalinosis, neutrophil count, lymphocyte count, and proteinuria) in model 3, the AUC for model 3 and model 4 (model 3 + RC) was 0.7564 and 0.7675, respectively. In the context of predicting mortality, the AUC was 0.6763 for model 1, 0.7304 for model 2, 0.8649 for model 3, and 0.8755 for model 4 (**Figures 4A**). To further analyze the prognostic values of risk factors, a nomogram model was constructed, incorporating all significant factors identified in the multivariate Cox regression analysis (**Figures 4C**). Each prediction parameter in the nomogram corresponded to specific values, which were summed and placed on the total score scale to calculate the survival probability. The nomogram illustrated age, arteriosclerosis, and RC as risk predictors of progression to CVD. Similarly, age, albumin, lymphocyte count, proteinuria, arteriosclerosis, and RC were identified as valuable predictors for all-cause mortality.

## 4 Discussion

In our study, we thoroughly examined the relationship between RC and both CVD events and mortality in patients with biopsy-proven DKD. Our investigation encompassed an in-depth analysis of the potential impact of RC on these critical clinical outcomes, shedding light on the intricate interplay between RC levels and the incidence of CVD events as well as all-cause mortality in this specific patient population.

Previous epidemiological study has established an association between traditional lipids and LV structure and function (32). However, the understanding of the relationships between RC levels and LV structure and function remains relatively limited. Notably, a prior study indicated that a high RC trajectory in young adults was associated with unfavorable alterations in LV structure and function (30). Consistent with previous observations, our study revealed that elevated RC levels in DKD patients were correlated with unfavorable heart structure and impaired systolic function. However, we did not observe an association between high RC levels and LV diastolic function, which contrasts with prior research. These inconsistencies may, in part, be attributed to population differences. RC may contribute to adverse CVD events by impacting LV structure and systolic function in DKD patients.

Several potential mechanisms can elucidate the relationship between RC and LV structure and function. Firstly, RC has the capacity to infiltrate the coronary artery intima and be phagocytosed by macrophages, leading to the formation of atherosclerotic plaques (33). This process reduces arterial elasticity, increases fragility, and gradually narrows or obstructs arterial lumens, resulting in cardiac ischemia and diminished contractile function. Furthermore, RC has been associated with arterial stiffness, which elevates LV load and prompts compensatory LV remodeling (34). Arterial stiffness increases LV load and leads to compensatory LV remodeling (30, 35). Additionally, RC may contribute to aortic stenosis and low-grade systemic inflammation, both of which are linked to LV remodeling (36, 37).

Emerging evidence underscores the independent and substantial contribution of RC to cardiovascular risk. For instance, in a post-trial cohort study involving older obese participants, individuals with RC levels equal to or exceeding 30 mg/dL faced a heightened risk of major adverse cardiovascular events (MACE), even if their LDL cholesterol levels had reached conventional targets (28). In a large U.S. community-based population, a high RC/low LDL-C phenotype was associated with increased ASCVD risk (38). In our subgroup analysis, we observed a significantly heightened risk of CVD events in individuals with elevated RC levels compared to those with lower RC levels among patients not utilizing statins. However, this association was not statistically significant in patients using statins. We posited that the use of statins might have attenuated the impact of RC in our study by influencing the occurrence of outcome events. In a prospective case-cohort study of individuals with newly diagnosed DKD, higher RC was associated with increased cardiovascular mortality, with the highest risk observed when elevated RC coexisted with higher LDL-C (39). This suggests a potential interplay between RC and LDL-C in the aforementioned patient cohort. Nonetheless, the precise mechanism underlying this interaction remains unclear. Irrespective of the existence of such an interaction, controlling RC may hold greater significance than further reducing LDL-C in individuals at risk for primary prevention who are ineligible for statin therapy or those already receiving high-dose statin therapy.

From a lipid-management standpoint, RC provides prognostic information beyond established atherogenic lipid markers, including LDL-C, non–high-density lipoprotein cholesterol (non-HDL-C), and apolipoprotein B (apoB) (38, 40). In individuals with renal insufficiency, LDL-C may be less informative for identifying excess ASCVD risk, whereas RC remains associated with risk; notably, elevated RC is often identifiable by higher non-HDL-C or apoB, supporting the incorporation of RC into routine lipid risk assessment rather than relying on LDL-C alone (41). In DM and CKD populations, RC also appears to capture residual renal risk despite attainment of conventional lipid targets. A nationwide cohort of newly diagnosed T2DM showed that higher RC was associated with incident CKD, even among participants meeting traditional lipid targets and studies in T2DM-related CKD further linked higher RC to adverse renal outcomes (42, 43). Consistently, a systematic review and meta-analysis concluded that RC is directly associated with a higher risk of CKD, supporting attention to RC in addition to traditional lipid markers in patients with or at risk for CKD (44). In conjunction with our findings, these data support a pragmatic refinement of lipid risk assessment for DKD: after achieving guideline-directed LDL-C targets (with consideration of non-HDL-C/apoB), elevated RC may identify patients with persistent atherosclerotic risk and justify closer follow-up and RC-focused risk reduction, rather than further LDL-C lowering alone.

Beyond risk stratification, RC is potentially modifiable. Because RC reflects cholesterol carried by TRL remnants, strategies that reduce TRL burden and remnant accumulation are expected to lower RC (45). Postprandial dyslipidemia is common in CKD and T2DM, and can be improved by lifestyle interventions (caloric restriction, weight reduction, and exercise) (46). Pharmacologic options that lower triglyceride-rich lipoproteins—such as fibrates, omega-3 fatty acids, and selected lipid- and glucose-lowering therapies—may also reduce remnant-related dyslipidemia, supporting the feasibility of RC reduction with existing approaches (46, 47). Emerging agents targeting TRL metabolism (e.g., apoC-III or ANGPTL3 inhibition) represent additional options, but require further evaluation in DKD populations before broad implementation (48, 49).

Our study meticulously examined the influence of gender as a baseline characteristic on outcome events. While our univariate Cox survival analysis did not reveal a direct association between gender and the incidence of outcome events, our subgroup analysis of female DKD patients indicated that those with higher RC concentrations faced an elevated risk of CVD events, a finding that did not reach statistical significance in men. Notably, CVD stood as the primary cause of death in women, constituting 35% of all female deaths in 2019 (50). A study conducted in 2001 identified RC as an independent risk factor for CVD in women (51).

This gender disparity may stem from practical considerations, as women were less likely to receive guideline-recommended lipid-lowering medications compared to men (52). Concurrently, women were also more inclined to decline initial treatment and exhibit lower adherence to prescribed regimens. Consequently, women may be more vulnerable to a lack of preventive management strategies and inadequate control of lipid levels. In light of these observations, our study suggests that reducing RC may represent an effective approach for managing cardiovascular risk in women, accounting for gender-specific differences. These findings may also offer insights for enhancing guidelines pertaining to CVD prevention and management.

In addition to investigating CVD events, our study delved into the association of RC with all-cause mortality, representing the first cohort study to explore potential links between RC and all-cause mortality in patients with DKD. Previous research has demonstrated that elevated RC levels are independently correlated with all-cause mortality in peritoneal dialysis patients after adjusting for confounding factors (53). Notably, below the saturation point of RC levels, the risk of all-cause mortality exhibited an upward trajectory with increasing RC levels. Another study, encompassing 5414 patients with ischemic heart disease, revealed a rise in the risk of all-cause mortality with increasing RC levels, an association not observed with escalating LDL-C levels, thereby implying that the impact of RC on all-cause mortality operates independently of LDL-C (54). Within our study, DKD patients with elevated RC levels exhibited heightened all-cause mortality. Consistent with prior research, the association between RC levels and all-cause mortality appeared more pronounced in women than in men within our study. While the precise mechanisms remain elusive, this discrepancy could be attributed to gender-specific variations in hormone and lipid metabolism. Furthermore, our observations indicated that the subgroup of patients not receiving statin therapy might be more susceptible to the adverse effects of RC. This susceptibility may stem from the therapeutic and preventive impacts of cardiovascular disease medications, with these protective effects potentially influencing the emergence of mortality outcomes.

### 4.1 Limitations

Several limitations of this study should be acknowledged. First, this single-center study enrolling Chinese patients with DKD confirmed by renal biopsy. The restricted source population may introduce selection bias and limit the generalizability of the findings. Second, owing to the study time window, the uptake of newer agents—including GLP-1 receptor agonists, SGLT2 inhibitors, and finerenone—was low in our cohort; therefore, the applicability of our results to the current treatment paradigm in which these agents play a central role may be limited. Third, because of constraints in the data collection protocol and availability, social determinants were not systematically captured at baseline, precluding a comprehensive assessment of their associations with RC levels and outcome risk. Fourth, as an observational study, despite multivariable adjustment, residual confounding cannot be fully excluded; the causal relationship between RC and cardiovascular outcomes, as well as all-cause mortality, requires further confirmation in prospective or interventional studies. Fifth, RC was estimated using a calculation-based approach, which is convenient and cost-efficient; however, discrepancies between calculated and directly measured RC may exist, potentially affecting effect estimates and leading to some overestimation of its clinical utility.

## 5 Conclusion

In conclusion, this study demonstrates that higher baseline RC levels are independently associated with an increased risk of CVD events and all-cause mortality among patients with DKD. These findings support the potential value of incorporating RC into risk stratification for DKD; however, the observational nature of this study precludes causal inference and the generalizability of the RC cutoff derived in this cohort requires external validation. Future multicenter prospective studies with larger sample sizes and longer follow-up are warranted to confirm these associations, to determine clinically meaningful RC thresholds, and to evaluate whether RC-guided preventive strategies can translate into reduced CVD events and mortality in DKD.

## Data Availability

The raw data supporting the conclusions of this article will be made available by the authors, without undue reservation.

## Supplementary material

Supplementary Figure 1 Flowchart of included patients in this study.

Supplementary Figure 2 The predictive cutoff value of the RC was evaluated using the time-dependent receiver operating characteristic (td-ROC) of the subjects. AUC, the area under the curve. **(A)** CVD events. **(B)** All-cause mortality.

Supplementary Figure 3 Kaplan-Meier curves for subgroup analysis in patients with biopsy-confirmed DKD with different types of clinical manifestations. **(A–H)** CVD events. **(I–P)** All-cause mortality.

Supplementary Table 1 Pathologic features of patients with biopsy-proven DKD across the tertiles of RC.

Supplementary Table 2 Univariate and multivariate Cox analyses on predictive value of medication use factors for endpoint events in DKD patients.

## References

[1] Selby NM Taal MW . An updated overview of diabetic nephropathy: Diagnosis, prognosis, treatment goals and latest guidelines. Diabetes Obes Metab. (2020) 22:3–15. doi: 10.1111/dom.14007, PMID: 32267079

[2] Martin SS Aday AW Almarzooq ZI Anderson CAM Arora P Avery CL . 2024 Heart disease and stroke statistics: A report of US and global data from the American heart association. Circulation. (2024) 149:e347–913. doi: 10.1161/CIR.0000000000001209, PMID: 38264914 PMC12146881

[3] Marx-Schütt K Cherney DZI Jankowski J Matsushita K Nardone M Marx N . Cardiovascular disease in chronic kidney disease. Eur Heart J. (2025) 46:2148–60. doi: 10.1093/eurheartj/ehaf167, PMID: 40196891 PMC12167664

[4] Sabanayagam C Chee ML Banu R Cheng C-Y Lim SC Tai ES . Association of diabetic retinopathy and diabetic kidney disease with all-cause and cardiovascular mortality in a multiethnic Asian population. JAMA Netw Open. (2019) 2:e191540. doi: 10.1001/jamanetworkopen.2019.1540, PMID: 30924904 PMC6450319

[5] Claudel SE Verma A . Albuminuria in cardiovascular, kidney, and metabolic disorders: A state-of-the-art review. Circulation. (2025) 151:716–32. doi: 10.1161/CIRCULATIONAHA.124.071079, PMID: 40063723 PMC11902889

[6] Ferreira JP Zannad F Butler J Filippatos G Pocock SJ Brueckmann M . Association of empagliflozin treatment with albuminuria levels in patients with heart failure: A secondary analysis of EMPEROR-pooled. JAMA Cardiol. (2022) 7:1148–59. doi: 10.1001/jamacardio.2022.2924, PMID: 36129693 PMC9494272

[7] Baaten CCFMJ Vondenhoff S Noels H . Endothelial cell dysfunction and increased cardiovascular risk in patients with chronic kidney disease. Circ Res. (2023) 132:970–92. doi: 10.1161/CIRCRESAHA.123.321752, PMID: 37053275 PMC10097498

[8] de Boer IH Khunti K Sadusky T Tuttle KR Neumiller JJ Rhee CM . Diabetes management in chronic kidney disease: A consensus report by the American diabetes association (ADA) and kidney disease: improving global outcomes (KDIGO). Diabetes Care. (2022) 45:3075–90. doi: 10.2337/dci22-0027, PMID: 36189689 PMC9870667

[9] Kidney Disease: Improving Global Outcomes (KDIGO) Diabetes Work Group . KDIGO 2020 clinical practice guideline for diabetes management in chronic kidney disease. Kidney Int. (2020) 98:S1–S115. doi: 10.1016/j.kint.2020.06.019, PMID: 32998798

[10] Ananda RA Gwini S Beilin LJ Schlaich MP Stowasser M Young MJ . Relationship Between renin, aldosterone, aldosterone-to-renin ratio and arterial stiffness and left ventricular mass index in young adults. Circulation. (2024) 150:2019–30. doi: 10.1161/CIRCULATIONAHA.124.070039, PMID: 39351674

[11] Noels H van der Vorst EPC Rubin S Emmett A Marx N Tomaszewski M . Renal-cardiac crosstalk in the pathogenesis and progression of heart failure. Circ Res. (2025) 136:1306–34. doi: 10.1161/CIRCRESAHA.124.325488, PMID: 40403103 PMC12105978

[12] Curaj A Vanholder R Loscalzo J Quach K Wu Z Jankowski V . Cardiovascular consequences of uremic metabolites: an overview of the involved signaling pathways. Circ Res. (2024) 134:592–613. doi: 10.1161/CIRCRESAHA.123.324001, PMID: 38422175

[13] Wang N Zhang C . Oxidative stress: A culprit in the progression of diabetic kidney disease. Antioxid (Basel). (2024) 13:455. doi: 10.3390/antiox13040455, PMID: 38671903 PMC11047699

[14] Ndumele CE Rangaswami J Chow SL Neeland IJ Tuttle KR Khan SS . Cardiovascular-kidney-metabolic health: A presidential advisory from the American heart association. Circulation. (2023) 148:1606–35. doi: 10.1161/CIR.0000000000001184, PMID: 37807924

[15] Wright AK Suarez-Ortegon MF Read SH Kontopantelis E Buchan I Emsley R . Risk factor control and cardiovascular event risk in people with type 2 diabetes in primary and secondary prevention settings. Circulation. (2020) 142:1925–36. doi: 10.1161/CIRCULATIONAHA.120.046783, PMID: 33196309 PMC7664968

[16] Chapman MJ Ginsberg HN Amarenco P Andreotti F Borén J Catapano AL . Triglyceride-rich lipoproteins and high-density lipoprotein cholesterol in patients at high risk of cardiovascular disease: evidence and guidance for management. Eur Heart J. (2011) 32:1345–61. doi: 10.1093/eurheartj/ehr112, PMID: 21531743 PMC3105250

[17] Varbo A Nordestgaard BG . Nonfasting triglycerides, low-density lipoprotein cholesterol, and heart failure risk: two cohort studies of 113 554 individuals. Arterioscler Thromb Vasc Biol. (2018) 38:464–72. doi: 10.1161/ATVBAHA.117.310269, PMID: 29097364

[18] Cosentino F Grant PJ Aboyans V Bailey CJ Ceriello A Delgado V . 2019 ESC Guidelines on diabetes, pre-diabetes, and cardiovascular diseases developed in collaboration with the EASD. Eur Heart J. (2020) 41:255–323. doi: 10.1093/eurheartj/ehz486, PMID: 31497854

[19] Twickler TB Dallinga-Thie GM Cohn JS Chapman MJ . Elevated remnant-like particle cholesterol concentration: a characteristic feature of the atherogenic lipoprotein phenotype. Circulation. (2004) 109:1918–25. doi: 10.1161/01.CIR.0000125278.58527.F3, PMID: 15117861

[20] AIM-HIGH Investigators Boden WE Probstfield JL Anderson T Chaitman BR Desvignes-Nickens P . Niacin in patients with low HDL cholesterol levels receiving intensive statin therapy. N Engl J Med. (2011) 365:2255–67. doi: 10.1056/NEJMoa1107579, PMID: 22085343

[21] Sascău R Clement A Radu R Prisacariu C Stătescu C . Triglyceride-rich lipoproteins and their remnants as silent promoters of atherosclerotic cardiovascular disease and other metabolic disorders: A review. Nutrients. (2021) 13:1774. doi: 10.3390/nu13061774, PMID: 34067469 PMC8224751

[22] Duran EK Aday AW Cook NR Buring JE Ridker PM Pradhan AD . Triglyceride-rich lipoprotein cholesterol, small dense LDL cholesterol, and incident cardiovascular disease. J Am Coll Cardiol. (2020) 75:2122–35. doi: 10.1016/j.jacc.2020.02.059, PMID: 32354380 PMC8064770

[23] Tall AR Thomas DG Gonzalez-Cabodevilla AG Goldberg IJ . Addressing dyslipidemic risk beyond LDL-cholesterol. J Clin Invest. (2022) 132:e148559. doi: 10.1172/JCI148559, PMID: 34981790 PMC8718149

[24] Nordestgaard BG Varbo A . Triglycerides and cardiovascular disease. Lancet. (2014) 384:626–35. doi: 10.1016/S0140-6736(14)61177-6, PMID: 25131982

[25] Jørgensen AB Frikke-Schmidt R West AS Grande P Nordestgaard BG Tybjærg-Hansen A . Genetically elevated non-fasting triglycerides and calculated remnant cholesterol as causal risk factors for myocardial infarction. Eur Heart J. (2013) 34:1826–33. doi: 10.1093/eurheartj/ehs431, PMID: 23248205

[26] Burnett JR Hooper AJ Hegele RA . Remnant cholesterol and atherosclerotic cardiovascular disease risk. J Am Coll Cardiol. (2020) 76:2736–9. doi: 10.1016/j.jacc.2020.10.029, PMID: 33272367

[27] Huh JH Han K Cho YK Roh E Kang JG Lee SJ . Remnant cholesterol and the risk of cardiovascular disease in type 2 diabetes: a nationwide longitudinal cohort study. Cardiovasc Diabetol. (2022) 21:228. doi: 10.1186/s12933-022-01667-6, PMID: 36324177 PMC9632127

[28] Castañer O Pintó X Subirana I Amor AJ Ros E Hernáez Á . Remnant cholesterol, not LDL cholesterol, is associated with incident cardiovascular disease. J Am Coll Cardiol. (2020) 76:2712–24. doi: 10.1016/j.jacc.2020.10.008, PMID: 33272365

[29] Jansson Sigfrids F Dahlström EH Forsblom C Sandholm N Harjutsalo V Taskinen M-R . Remnant cholesterol predicts progression of diabetic nephropathy and retinopathy in type 1 diabetes. J Intern Med. (2021) 290:632–45. doi: 10.1111/joim.13298, PMID: 33964025

[30] Xu X Wang Z Huang R Guo Y Xiong Z Zhuang X . Remnant cholesterol in young adulthood is associated with left ventricular remodeling and dysfunction in middle age: the CARDIA study. Circ Cardiovasc Imaging. (2023) 16:e015589. doi: 10.1161/CIRCIMAGING.123.015589, PMID: 37988449 PMC10659242

[31] Tervaert TWC Mooyaart AL Amann K Cohen AH Cook HT Drachenberg CB . Pathologic classification of diabetic nephropathy. J Am Soc Nephrol. (2010) 21:556–63. doi: 10.1681/ASN.2010010010, PMID: 20167701

[32] de las Fuentes L Waggoner AD Brown AL Dávila-Román VG . Plasma triglyceride level is an independent predictor of altered left ventricular relaxation. J Am Soc Echocardiogr. (2005) 18:1285–91. doi: 10.1016/j.echo.2005.05.002, PMID: 16376756

[33] Wadström BN Pedersen KM Wulff AB Nordestgaard BG . Elevated remnant cholesterol, plasma triglycerides, and cardiovascular and non-cardiovascular mortality. Eur Heart J. (2023) 44:1432–45. doi: 10.1093/eurheartj/ehac822, PMID: 36631967

[34] Ohyama Y Ambale-Venkatesh B Noda C Chugh AR Teixido-Tura G Kim J-Y . Association of aortic stiffness with left ventricular remodeling and reduced left ventricular function measured by magnetic resonance imaging: the multi-ethnic study of atherosclerosis. Circ Cardiovasc Imaging. (2016) 9:e004426. doi: 10.1161/CIRCIMAGING.115.004426, PMID: 27353852 PMC4933322

[35] Kaltoft M Langsted A Nordestgaard BG . Triglycerides and remnant cholesterol associated with risk of aortic valve stenosis: Mendelian randomization in the Copenhagen General Population Study. Eur Heart J. (2020) 41:2288–99. doi: 10.1093/eurheartj/ehaa172, PMID: 32267934

[36] Li Z-H Hao Q-Y Zeng Y-H Guo J-B Li S-C Gao J-W . Remnant cholesterol and the risk of aortic valve calcium progression: insights from the MESA study. Cardiovasc Diabetol. (2024) 23:20. doi: 10.1186/s12933-023-02081-2, PMID: 38195550 PMC10777602

[37] Kraaijenhof JM Kerkvliet MJ Nurmohamed NS Grefhorst A Kroon J Wareham NJ . The role of systemic inflammation in remnant cholesterol associated cardiovascular risk: insights from the EPIC-Norfolk study. Eur J Prev Cardiol. (2025) 6:zwaf037. doi: 10.1093/eurjpc/zwaf037, PMID: 39910741

[38] Quispe R Martin SS Michos ED Lamba I Blumenthal RS Saeed A . Remnant cholesterol predicts cardiovascular disease beyond LDL and ApoB: a primary prevention study. Eur Heart J. (2021) 42:4324–32. doi: 10.1093/eurheartj/ehab432, PMID: 34293083 PMC8572557

[39] Yu D Wang Z Zhang X Qu B Cai Y Ma S . Remnant cholesterol and cardiovascular mortality in patients with type 2 diabetes and incident diabetic nephropathy. J Clin Endocrinol Metab. (2021) 106:3546–54. doi: 10.1210/clinem/dgab533, PMID: 34291804

[40] Hansen MK Mortensen MB Warnakula Olesen KK Thrane PG Maeng M . Non-HDL cholesterol and residual risk of cardiovascular events in patients with ischemic heart disease and well-controlled LDL cholesterol: a cohort study. Lancet Reg Health Eur. (2024) 36:100774. doi: 10.1016/j.lanepe.2023.100774, PMID: 38019978 PMC10652132

[41] Elías-López D Vedel-Krogh S Kobylecki CJ Wadström BN Nordestgaard BG . Impaired renal function with higher remnant cholesterol related to risk of atherosclerotic cardiovascular disease: A cohort study. Arterioscler Thromb Vasc Biol. (2024) 44:2647–58. doi: 10.1161/ATVBAHA.124.321387, PMID: 39445427

[42] Jang SY Kang M Song E Jang A Choi KM Baik SH . Remnant cholesterol is an independent risk factor for the incidence of chronic kidney disease in newly-diagnosed type 2 diabetes: A nationwide population-based study. Diabetes Res Clin Pract. (2024) 210:111639. doi: 10.1016/j.diabres.2024.111639, PMID: 38548106

[43] Li Q Wang T Shao X Fan X Lin Y Cui Z . Association of remnant cholesterol with renal function and its progression in patients with type 2 diabetes related chronic kidney disease. Front Endocrinol (Lausanne). (2024) 15:1331603. doi: 10.3389/fendo.2024.1331603, PMID: 39027471 PMC11254661

[44] Karakasis P Patoulias D Rizzo M Fragakis N Mantzoros CS . Association between remnant cholesterol and chronic kidney disease: Systematic review and meta-analysis. Diabetes Obes Metab. (2025) 27:2573–83. doi: 10.1111/dom.16258, PMID: 39950216 PMC11964997

[45] Ginsberg HN Packard CJ Chapman MJ Borén J Aguilar-Salinas CA Averna M . Triglyceride-rich lipoproteins and their remnants: metabolic insights, role in atherosclerotic cardiovascular disease, and emerging therapeutic strategies-a consensus statement from the European Atherosclerosis Society. Eur Heart J. (2021) 42:4791–806. doi: 10.1093/eurheartj/ehab551, PMID: 34472586 PMC8670783

[46] Virani SS Morris PB Agarwala A Ballantyne CM Birtcher KK Kris-Etherton PM . 2021 ACC expert consensus decision pathway on the management of ASCVD risk reduction in patients with persistent hypertriglyceridemia: A report of the american college of cardiology solution set oversight committee. J Am Coll Cardiol. (2021) 78:960–93. doi: 10.1016/j.jacc.2021.06.011, PMID: 34332805

[47] Das Pradhan A Glynn RJ Fruchart J-C MacFadyen JG Zaharris ES Everett BM . Triglyceride lowering with pemafibrate to reduce cardiovascular risk. N Engl J Med. (2022) 387:1923–34. doi: 10.1056/NEJMoa2210645, PMID: 36342113

[48] Zimerman A Wiviott SD Park J-G Murphy SA Ran X Bramson CR . Reductions in remnant cholesterol and VLDL cholesterol through inhibition of ANGPTL3 protein synthesis: an analysis from the TRANSLATE-TIMI 70 trial. Eur J Prev Cardiol. (2024) 31:1216–23. doi: 10.1093/eurjpc/zwae090, PMID: 38484368

[49] Bergmark BA Marston NA Prohaska TA Alexander VJ Zimerman A Moura FA . Olezarsen for hypertriglyceridemia in patients at high cardiovascular risk. N Engl J Med. (2024) 390:1770–80. doi: 10.1056/NEJMoa2402309, PMID: 38587249

[50] Cho L Davis M Elgendy I Epps K Lindley KJ Mehta PK . Summary of updated recommendations for primary prevention of cardiovascular disease in women: JACC state-of-the-art review. J Am Coll Cardiol. (2020) 75:2602–18. doi: 10.1016/j.jacc.2020.03.060, PMID: 32439010 PMC8328156

[51] McNamara JR Shah PK Nakajima K Cupples LA Wilson PW Ordovas JM . Remnant-like particle (RLP) cholesterol is an independent cardiovascular disease risk factor in women: results from the Framingham Heart Study. Atherosclerosis. (2001) 154:229–36. doi: 10.1016/s0021-9150(00)00484-6, PMID: 11137104

[52] Nanna MG Wang TY Xiang Q Goldberg AC Robinson JG Roger VL . Sex differences in the use of statins in community practice. Circ Cardiovasc Qual Outcomes. (2019) 12:e005562. doi: 10.1161/CIRCOUTCOMES.118.005562, PMID: 31416347 PMC6903404

[53] Deng J Tang R Chen J Zhou Q Zhan X Long H . Remnant cholesterol as a risk factor for all-cause and cardiovascular mortality in incident peritoneal dialysis patients. Nutr Metab Cardiovasc Dis. (2023) 33:1049–56. doi: 10.1016/j.numecd.2023.02.009, PMID: 36948938

[54] Jepsen A-MK Langsted A Varbo A Bang LE Kamstrup PR Nordestgaard BG . Increased remnant cholesterol explains part of residual risk of all-cause mortality in 5414 patients with ischemic heart disease. Clin Chem. (2016) 62:593–604. doi: 10.1373/clinchem.2015.253757, PMID: 26888894

