# Supplementary figures and images for: Association of remnant cholesterol with cardiovascular events and mortality in biopsy-proven diabetic kidney disease
Source: Front Endocrinol (Lausanne). 2026 Jan 28;17:1720189. doi: 10.3389/fendo.2026.1720189 (PMC12890626; doi:10.3389/fendo.2026.1720189)

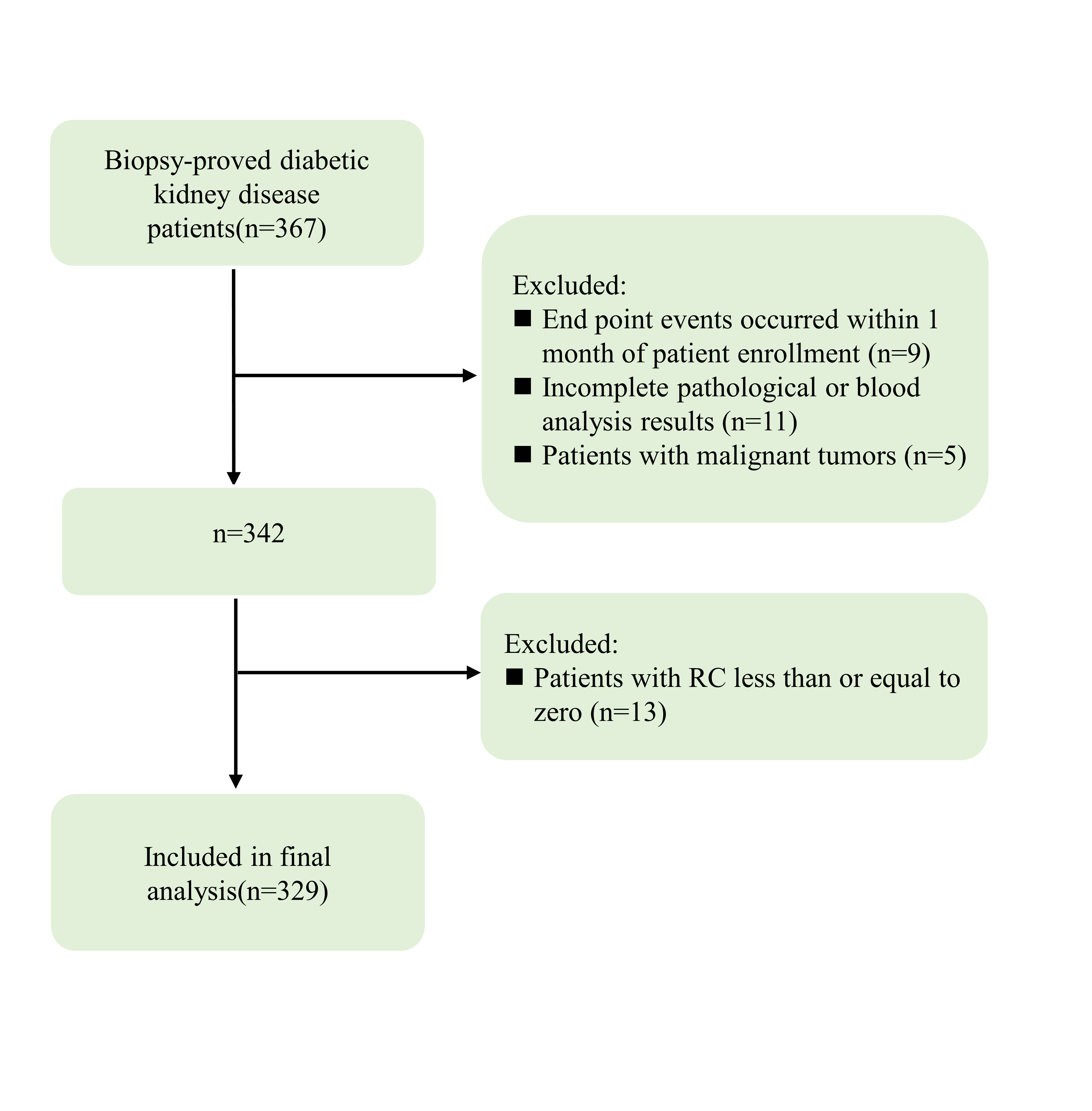

Supplement: Supplementary Figure 1 — Flowchart of included patients in this study. [file Image1.tif]

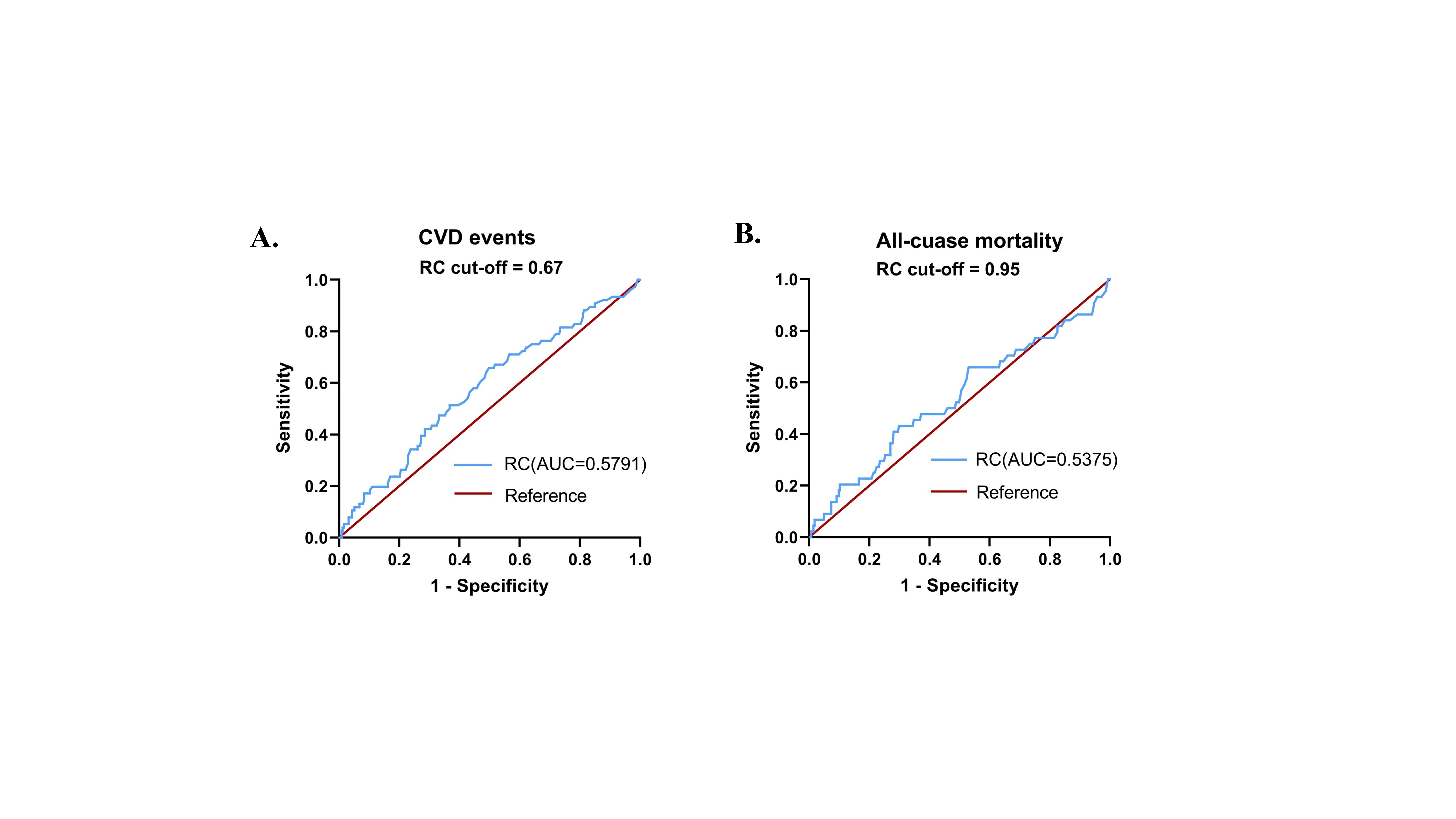

Supplement: Supplementary Figure 2 — The predictive cutoff value of the RC was evaluated using the time-dependent receiver operating characteristic (td-ROC) of the subjects. AUC, the area under the curve. (A) CVD events. (B) All-cause mortality. [file Image2.tif]

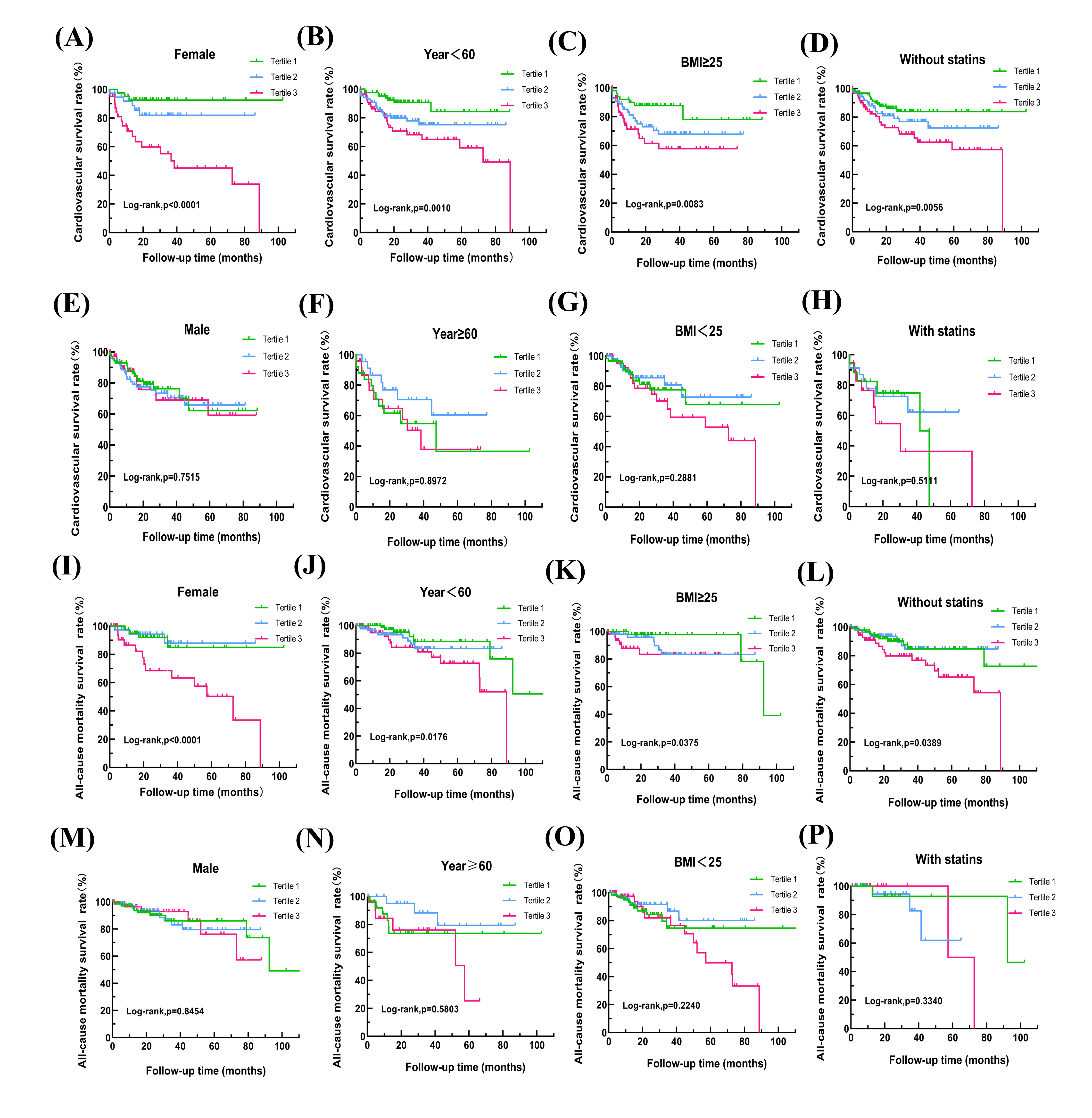

Supplement: Supplementary Figure 3 — Kaplan-Meier curves for subgroup analysis in patients with biopsy-confirmed DKD with different types of clinical manifestations. (A–H) CVD events. (I–P) All-cause mortality. [file Image3.tif]
